# CCDC34 is up-regulated in bladder cancer and regulates bladder cancer cell proliferation, apoptosis and migration

**DOI:** 10.18632/oncotarget.4624

**Published:** 2015-07-16

**Authors:** Yanqing Gong, Wei Qiu, Xianghui Ning, Xinyu Yang, Libo Liu, Zicheng Wang, Jian Lin, Xuesong Li, Yinglu Guo

**Affiliations:** ^1^ Department of Urology, Peking University First Hospital, Beijing 100034, China; ^2^ Institute of Urology, Peking University, Beijing 100034, China; ^3^ National Urological Cancer Center, Beijing 100034, China; ^4^ Department of Urology, Beijing Friendship Hospital, Capital Medical University, Beijing 100050, China

**Keywords:** CCDC34, bladder cancer, siRNA, proliferation, migration

## Abstract

The coiled coil is a superhelical structural protein motif involved in a diverse array of biological functions, and the abnormal expression of the coiled-coil domain containing proteins has a direct link with the phenotype of tumor cell migration, invasion and metastasis. The aim of this study was to investigate the critical role of Coiled-coil domain-containing protein 34 (CCDC34) in bladder carcinogenesis, which has never been reported to date. Here, we found CCDC34 expression was elevated in bladder cancer tissues and cell lines. The knockdown of CCDC34 via lentivirus-mediated siRNA significantly suppressed bladder cancer cells proliferation and migration, and induced cell cycle arrest at G2/M phase and increased apoptosis *in vitro*. In addition, CCDC34 knockdown suppressed bladder tumor growth in nude mice. Moreover, CCDC34 silencing decreased the phosphorylation of MEK, ERK1/2, JNK, p38 and Akt, and the expressions of c-Raf and c-Jun, indicating MAPK and AKT pathways (ERK/MAPK, p38/MAPK, JNK/MAPK and PI3K/Akt) might be involved in CCDC34 regulation of bladder cancer cell proliferation and migration. Our findings revealed for the first time a potential oncogenic role for CCDC34 in bladder carcinoma pathogenesis and it may serve as a biomarker or even a therapeutic target for bladder cancer.

## INTRODUCTION

Urothelial carcinoma of the bladder is a major cause of morbidity and mortality worldwide, with 180, 500 estimated new cases each year and 38, 200 deaths in the European Union [1], and with 74,000 estimated new cases and 16,000 deaths in United States [2]. Although localized bladder carcinomas could be managed by surgical resection, the recurrence and progression rates are still high. The therapeutic outcomes for patients with advanced bladder carcinoma who receive radiotherapy or chemotherapy remain unsatisfactory. The absence of more effective therapies for bladder carcinoma requires more research into the underlying molecular mechanisms of its tumorigenesis and the development of new treatment aimed at specific molecular targets.

Mitogen-activated protein kinase (MAPK) cascades are key signaling pathways involved in multiple biologic processes, such as cell proliferation, differentiation, death, migration, invasion and inflammation. Activation of MAPK is a frequent event in tumor progression and metastasis. Several components of the MAPK network have already been proposed as targets in cancer therapies, such as p38, JNK, ERK, MEK, RAF, RAS, and DUSP1. Over-activation of Ha-ras occurred in human urothelial tumors and hyper-activation of the ras signaling pathway was responsible for the low-grade, non-invasive papillary bladder tumors [3]. The p38 MAPK was activated during the log phase growth of bladder cancer cells and regulated invasion of bladder cancer by modulation of MMP-2 and MMP-9 expression and activity [4]. Besides, PI3K/AKT/mTOR is also a major intracellular signaling pathway, which is frequently activated in diverse cancers and plays a very significant role in cell growth, tumorigenesis, cell invasion and drug response. Elevated levels of active Akt have been proposed to mediate resistance to the pro-apoptotic cytokine tumor necrosis factor-related apoptosis-inducing ligand (TRAIL) in bladder cancer cells and were reversible upon PI3K inhibition [5]. Angiogenin (ANG) interacted with ribonuclease inhibitor and ANG up-regulation activated phosphorylation of key downstream target molecules of PI3K/AKT/mTOR signaling pathway, leading to the promotion of tumor angiogenesis, tumorigenesis and metastasis in bladder cancer cells [6].

The coiled-coil domain is a structural motif found in proteins that are involved in a diverse array of biological functions such as the regulation of gene expression, cell division, membrane fusion and drug extrusion and delivery [7]. The abnormal expression of the coiled-coil domain containing proteins in nasopharyngeal carcinoma [8], gastric cancer [9–10], prostate cancer [11], pancreatic cancer [12], breast cancer [13], colorectal cancer [14–15], has a direct link with the phenotype of tumor cell migration, invasion and metastasis. CCDC34, also known as Renal carcinoma antigen NY-REN-41, is a protein-coding and disease related gene. It consists of 373 amino acids and its chromosomal location is 11p14.1. Chromosome 11 is one of the most gene- and disease-rich chromosomes in the human genome. A chromosomal aberration involving CCDC34 (Translocation t(11;18) (p13;p11.2)) is found in a patient with hamartoma of the retinal pigment epithelium and retina [16]. However, the biological and clinical significances of CCDC34 in human bladder carcinoma remain largely unknown, prompting us to examine CCDC34 functional roles in bladder carcinoma pathogenesis.

In this study, we examined the expression of CCDC34 in human bladder cancer tissues and cell lines. We used lentivirus-mediated specific siRNA targeting of CCDC34 to investigate the role of its silencing on the proliferation, migration and cell cycle progression of bladder cancer cells. We also explored intratumor delivery of CCDC34 siRNA on xenograft tumorigenesis. Further analysis revealed that significant inactivation of the MAPK and AKT pathways might be involved in CCDC34 regulation of bladder cancer cell proliferation and migration. Our findings revealed for the first time a potential oncogenic role for CCDC34 in bladder cancer and it might be a useful target for bladder cancer therapy.

## RESULTS

### CCDC34 is up-regulated in bladder cancer tissues and cell lines

**Figure 1:**
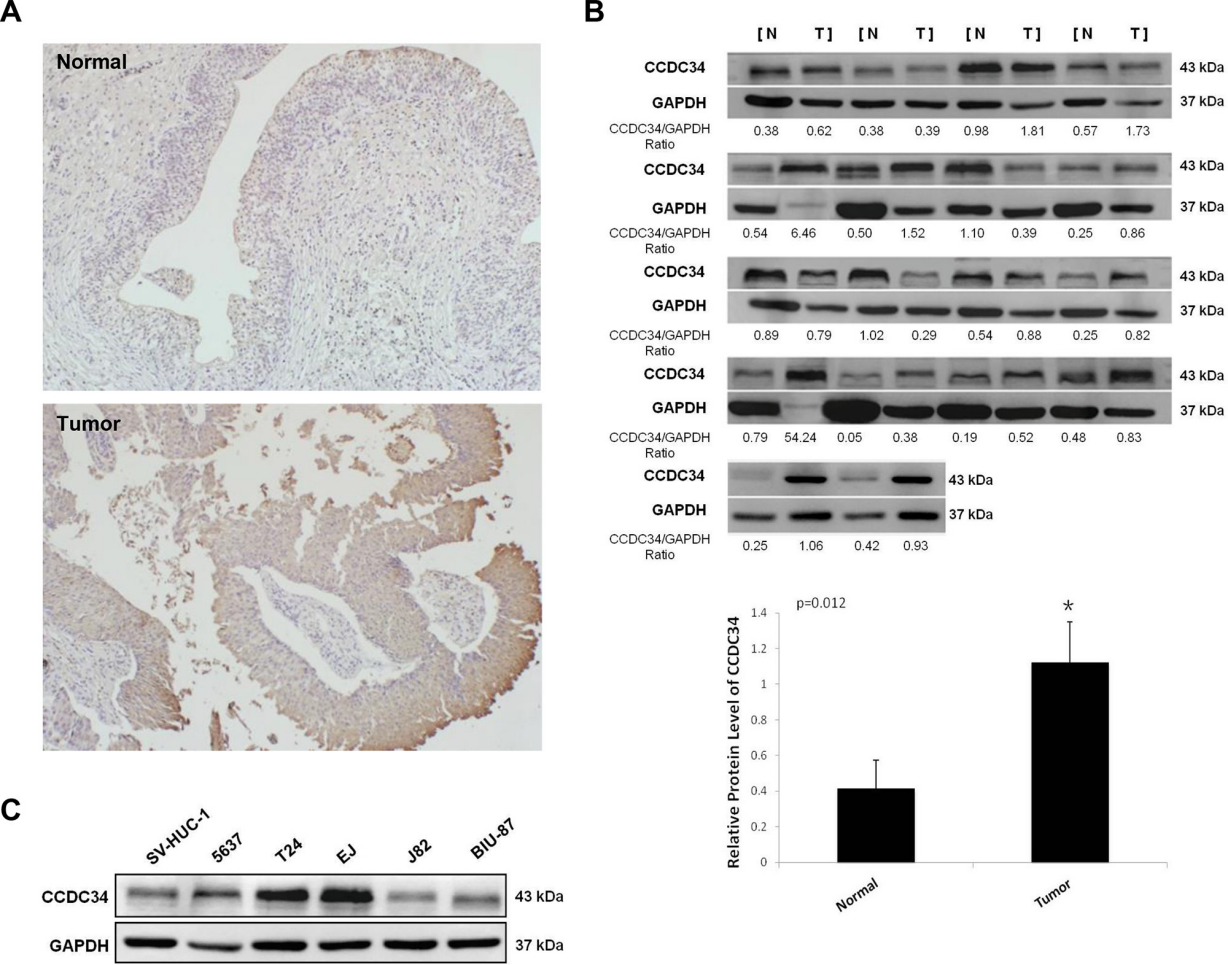
Increased expression of CCDC34 in human bladder cancer **A.** Representative immunohistochemistry staining of CCDC34 in normal control tissues and bladder cancer tissues (×100). **B.** Western blots showing the expression of CCDC34 in bladder cancer tissues (Tumor) and paraneoplastic bladder tissues (Normal). The ratios of CCDC34 band densities to relative GAPDH loading control are shown. Relative CCDC34 protein expression was expressed as mean ± SD from 18 cases, *P < 0.05. **C.** Western blots showing the expression of CCDC34 in human bladder cancer cells. Normal human urinary tract epithelial cell line SV-HUC-1 was used as negative control. GAPDH served as loading control.

We examined CCDC34 expression in 42 pairs of bladder urothelial carcinoma and matched adjacent normal tissues by immunohistochemical staining. CCDC34 was distributed mainly in the cytoplasm. The results showed weak staining in normal bladder tissues, while strong staining of CCDC34 was detected in almost all the cancer specimens (Fig. 1A). To confirm the results, we performed Western blot to detect CCDC34 expression in 18 bladder cancer specimens which were diagnosed by histopathological examination. The results showed that CCDC34 expressed higher level in bladder cancer specimens than in the paraneoplastic normal bladder tissues (*P* = 0.012, Fig. 1B).

Next, we performed Western blot to detect CCDC34 expression in the bladder cancer cell lines 5637, T24, J82, EJ and BIU-87, and the normal human urinary tract epithelial cell line SV-HUC-1. CCDC34 expression was higher in bladder cancer cell lines than in SV-HUC-1. Of the bladder cancer cell lines, T24, 5637 and EJ showed the highest CCDC34 expression (Fig. 1C). Thus, we chose T24 and 5637 for further functional characterization.

### Knockdown of CCDC34 inhibited bladder cancer cell growth *in vitro* and *in vivo*

**Figure 2:**
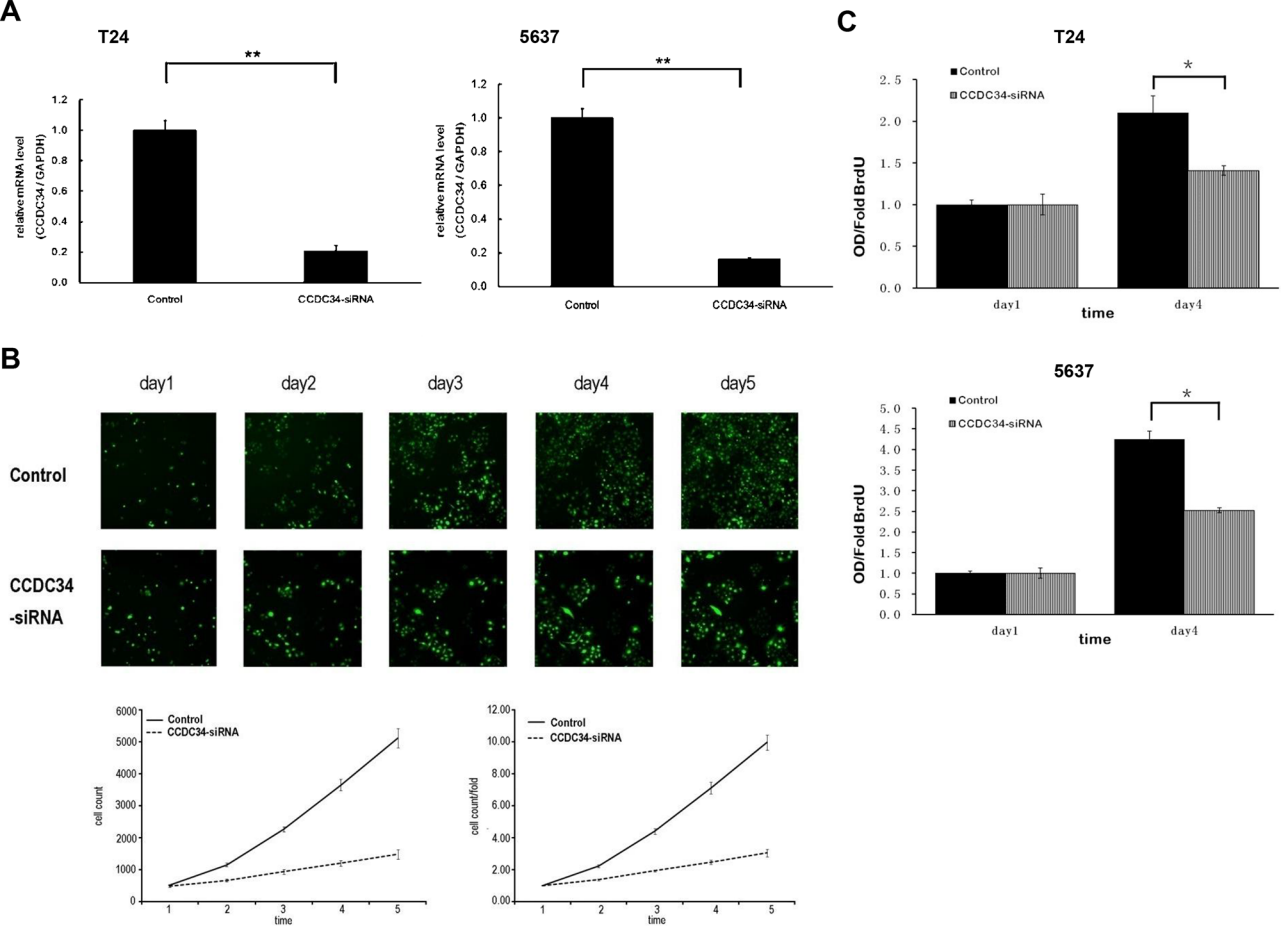
Lentivirus-mediated knockdown of CCDC34 inhibited human bladder cancer cell growth **A.** Quantitative RT-PCR revealed the CCDC34 expression was efficiently knockdown in T24 and 5637 cells. **P < 0.01. Control, cells infected with negative control lentivirus (NC lentiviral vectors); CCDC34-siRNA, cells infected with CCDC34-siRNA lentivirus. **B.** CCDC34 knockdown attenuated T24 cells growth potential *in vitro*. Cell growth was measured via multiparametric high-content screening (HCS) every day for five days. Data are shown as mean ± SD. **C.** The DNA synthesis rate was slowed down with CCDC34 knockdown, analyzed by BrdU incorporation assay on the 1st and 4^th^ days. Data are shown as mean ± SD, Control vs CCDC34-siRNA, *P < 0.05.

To investigate the mechanism by which CCDC34 contributes to malignancy of bladder cancer, we did Lentivirus-mediated knockdown of CCDC34 in T24 and 5637 cells. The lentivirus infection efficiency is above 85% for both CCDC34-siRNA lentivirus and Negative Control lentivirus, so that we can ensure the synchronization of all the following experiments (Supplemental Fig 1). CCDC34 mRNA levels were assessed by quantitative RT-PCR. The results showed CCDC34-siRNA lentivirus infected cultures exhibited significantly reduced CCDC34 transcripts compared with cells infected with NC lentivirus (*P* < 0.01, Fig. 2A).

In order to explore the function of CCDC34 on cell growth, T24 cells expressing either CCDC34 -siRNA lentivirus or NC lentivirus were seeded in 96-well plates, and cell growth was monitored by high-content screening (HCS) every day for 5 days. Cell growth rate was defined as: cell count of Nth day/cell count of 1st day, where *n* = 2, 3, 4, 5. The results showed that down-regulation of CCDC34 decreased the total number of cells and cell growth rate was slowed down (Table 1 and Fig. 2B). The DNA synthesis was also analyzed by BrdU incorporation assay on the 1st and 4th days in T24 and 5637 cells. The results showed decreased DNA synthesis in CCDC34-siRNA lentivirus infected group, indicating cell proliferation was significantly slowed down over the course of 4 days (0.01 < *P* < 0.05, Fig. 2C).

**Table 1:** Cell numbers and growth rate counted by cellomics

Time(Day)	Cell count		Cell count/Fold	
	Control	CCDC34-siRNA	Control	CCDC34-siRNA
Day 1	513.3 ± 13.05	481.0 ± 49.51	1.00 ± 0.00	1.00 ± 0.00
Day 2	1143.3 ± 58.79	656.7 ± 38.00	2.23 ± 0.09	1.37 ± 0.08
Day 3	2258.3 ± 76.54	928.3 ± 124.03	4.40 ± 0.17	1.93 ± 0.08
Day 4	3646.3 ± 176.19	1192.7 ± 206.63	7.11 ± 0.37	2.47 ± 0.19
Day 5	5113.0 ± 295.13	1474.3 ± 272.46	9.96 ± 0.47	3.05 ± 0.25

Cell count/Fold (cell growth rate) was defined as: cell count of Nth day/cell count of 1st day, where *n* = 2, 3, 4, 5.

**Figure 3:**
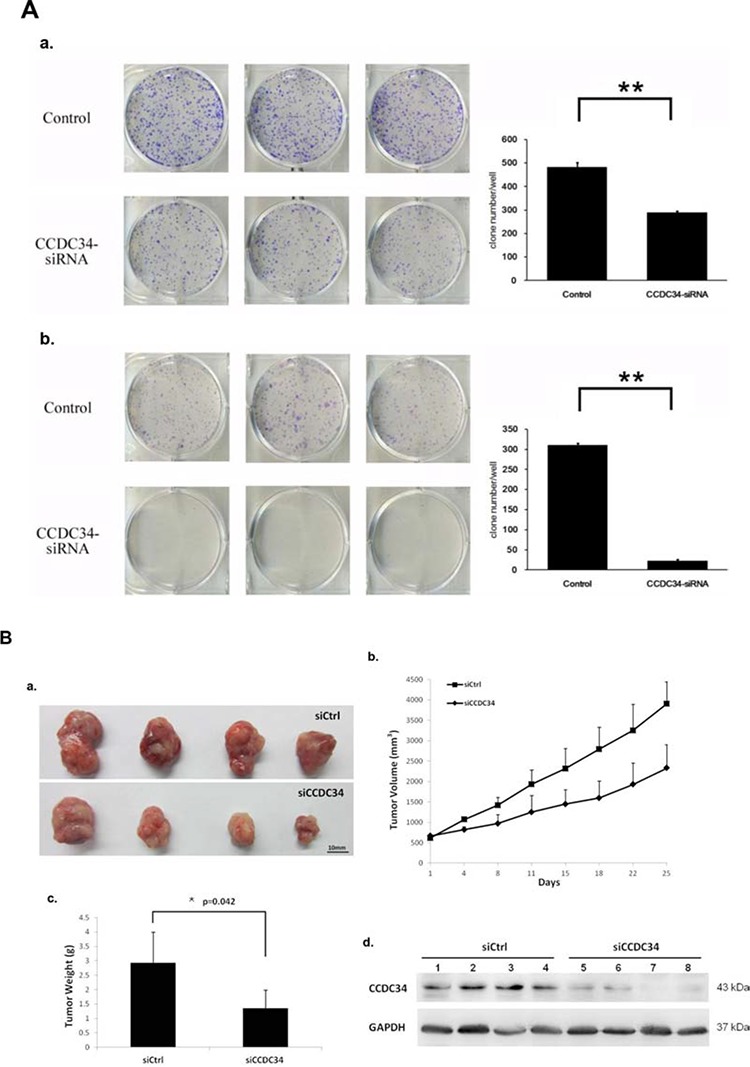
The growth-suppressive effect of CCDC34 knockdown on bladder cancer cells *in vitro* and *in vivo* **A.** CCDC34 knockdown significantly reduced colony formation in T24 (a) and 5637 (b) cells, as assessed by colony formation assay. Data are presented as mean ± SEM from three independent experiments with each running in triplicate. **P < 0.01. **B.** Therapeutic CCDC34 siRNA reduced the tumorigenicity of T24 xenograft *in vivo*. a, b and c, CCDC34 siRNA significantly decreased tumor vs control (siCtrl) treatment. a, Representative pictures of tumors were shown (*n* = 6); b, size; c, weight; d, CCDC34 expression was significantly reduced *in vivo*.

Furthermore, we assayed the colony formation to determine CCDC34 knockdown in bladder cancer cell tumorigenesis *in vitro*. The results showed that CCDC34 knockdown in both T24 and 5637 cells drastically caused a substantial reduction in colony formation compared with the control cells. Number of colonies in CCDC34-siRNA lentivirus infected cells was statistically less than in negative control groups, with Control 482 ± 21 vs. CCDC34-siRNA 289 ± 7 in T24 cells and Control 310 ± 5 vs. CCDC34-siRNA 22 ± 4 in 5637 cells (*P* < 0.01, Fig. 3A). Next, we investigated the effect of therapeutic CCDC34 siRNA on tumor growth *in vivo*. The T24 xenograft tumor taken rate in nude mice is 100%, and then the nude mice were randomly selected and treated with CCDC34 siRNA (siCCDC34) or control siRNA (siCtrl) twice a week for 4 weeks by intratumor injection. Mean T24 tumor volume and body weight at baseline were similar in the two groups, tumor volume (mm^3^): siCtrl 616.6 ± 27 vs. siCCDC34 656.7 ± 20; body weight (g): siCtrl 19.25 ± 3.4 vs. siCCDC34 19.97 ± 3.1. CCDC34 siRNA treatment significantly decreased tumor growth, as shown by strongly reduced tumor size and weight compared with the control treatment (Fig. 3B a, 3B b, 3B c). To confirm whether CCDC34 was knocked down *in vivo* by its siRNA injection, we also measured CCDC34 expression and found that it was significantly decreased in treated xenograft tumors (Fig. 3B d). These results indicated that CCDC34 is critical for bladder cancer cell proliferation and tumorigenicity.

### Knockdown of CCDC34 inhibited bladder cancer cell migration *in vitro*

**Figure 4:**
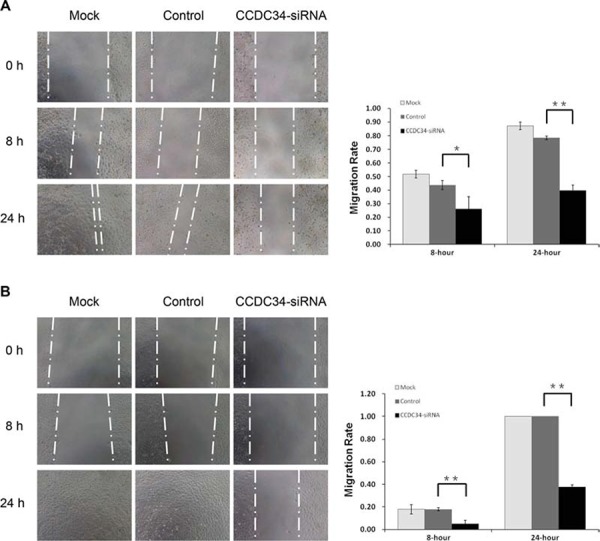
CCDC34 knockdown slowed the wound healing in T24 and 5637 cells Image acquisition of wound fields was done after removal of inserts (0 hours) and wound closure documentation was done at 0, 8 and 24 hours (h). Mock, non-infected control cells; Control, cells infected with NC lentivirus; CCDC34-siRNA, cells infected with CCDC34-siRNA lentivirus. **A.** T24 cells. **B.** 5637 cells. Migration rate was defined as: migration distances/width of open wound at 0 hour. Data are shown as mean ± SD, **P < 0.01.

*In vitro* wound healing assay was performed to evaluate the influence of CCDC34 on bladder cancer cell migration. After 24 hours, Microscopic analysis of the non-infected T24/5637 cells showed an almost complete closure of the gap, while CCDC34 knockdown cells closed the wound much slower, indicating the lowest migratory ability compared with non-infected cells and cells infected with NC lentivirus (Fig. 4A&4B), which might be partially due to the results of decreased proliferation.

### Knockdown of CCDC34 induced cell cycle arrest and apoptosis

**Figure 5:**
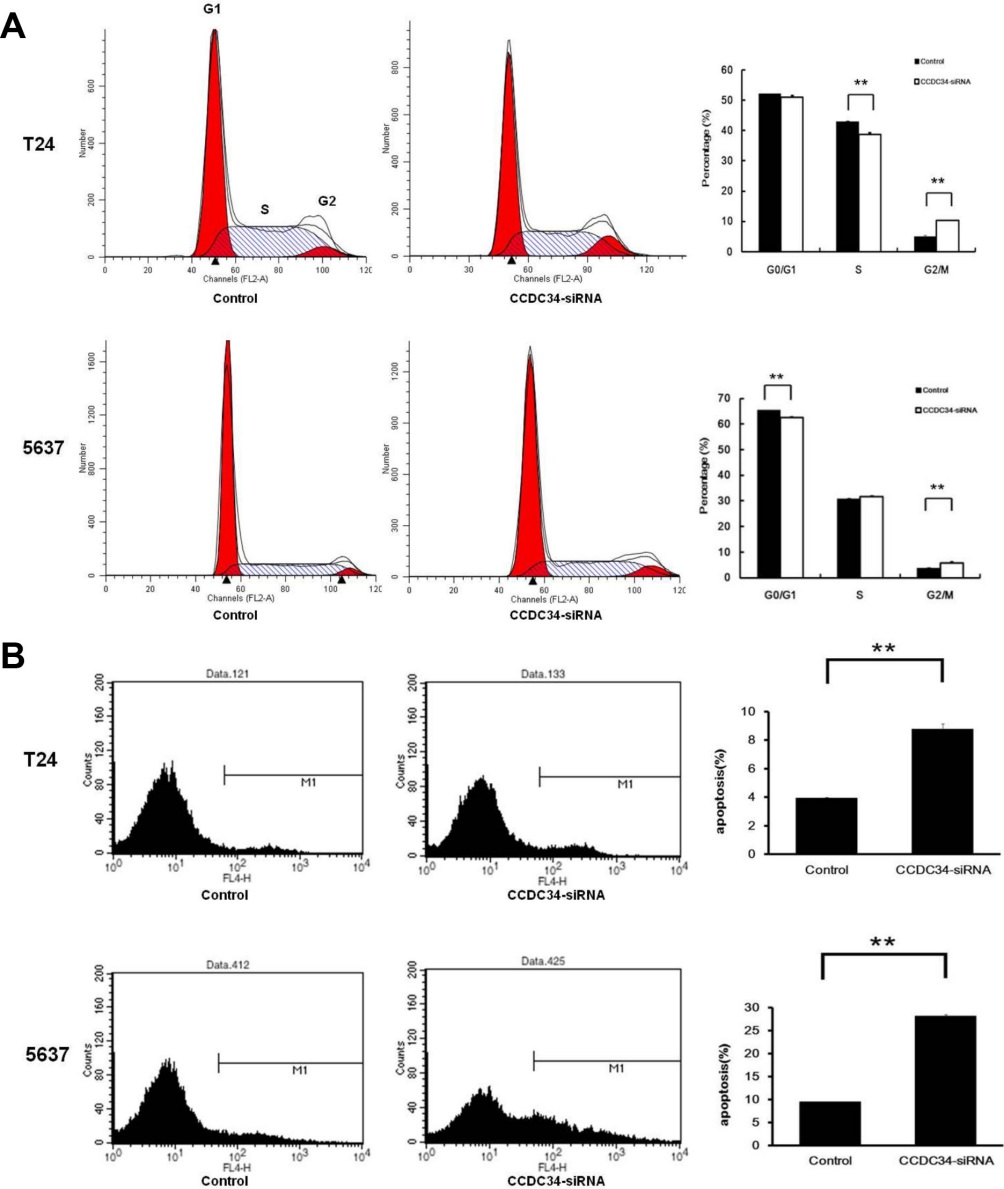
CCDC34 knockdown induced cell cycle arrest and apoptosis in T24 and 5637 cells **A.** Knockdown of CCDC34 expression induced G2/M phase arrest. **B.** Knockdown of CCDC34 induced increased cell apoptosis. Data are shown as mean ± SD, **P < 0.01.

Cell proliferative alteration is usually caused by changing cell cycle or apoptosis. To further explore these mechanisms, we examined the cell cycle by PI/FACS and detected apoptosis by Annexin V/FACS. As shown in Fig. 5A, for T24 cells, the Control group displayed the following distribution: (G0/G1 52.2%, S 42.87%, G2/M 4.88%), and the CCDC34-siRNA group displayed the following: (G0/G1 51.04%, S 38.68%, G2/M 10.28%). For 5637 cells, the Control group displayed the following distribution: (G0/G1 65.5%, S 30.92%, G2/M 3.56%), and the CCDC34-siRNA group displayed the following: (G0/G1 62.6%, S 31.26%, G2/M 5.76%). Compared with the Control group, CCDC34-siRNA group displayed a significant decrease in S phase or G0/G1 phase in the percentage of T24 and 5637 cells respectively; and a significant increase in G2/M phase for both cell lines, suggesting that cells were arrested in G2/M phase after CCDC34 gene silencing and CCDC34 gene was significantly correlated with cell cycle distribution (*P* < 0.01, Fig. 5A).

Moreover, as shown in Fig. 5B, the percentage of T24 cells in apoptosis phase was significantly increased in the CCDC34-siRNA group compared with Control group (Control 3.91 ± 0.08% vs. CCDC34-siRNA 8.77 ± 0.38%, *P* = 0.001). The percentage of 5637 cells in apoptosis phase was also significantly increased in the CCDC34-siRNA group compared with Control group (Control 9.48 ± 0.05% vs. CCDC34-siRNA 27.86 ± 0.39%, *P* = 0.0001). These results affirmed that CCDC34 may be related with the apoptosis of bladder cancer cells.

### CCDC34 knockdown suppressed activation of MAPK and AKT signaling pathways

**Figure 6:**
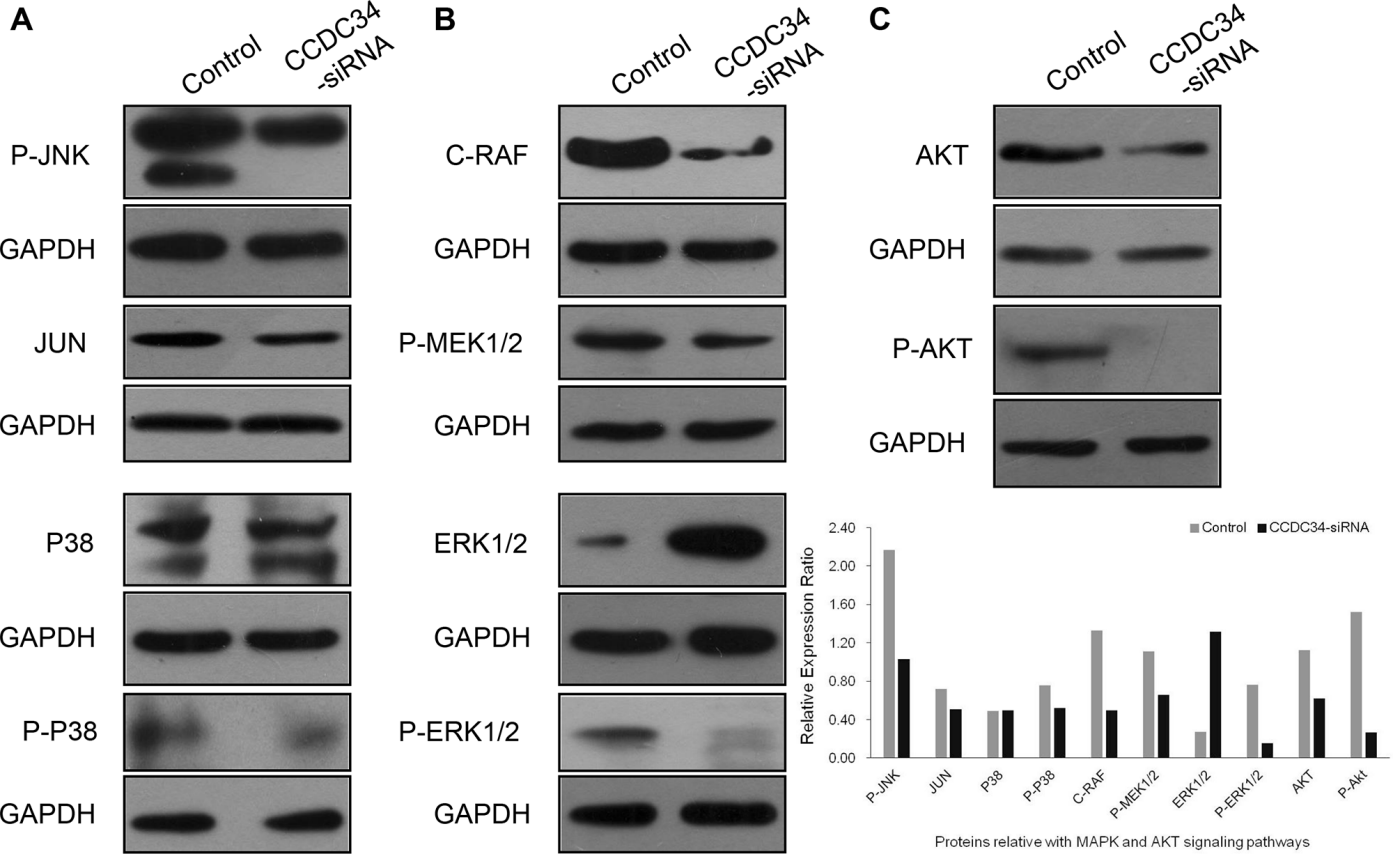
CCDC34 knockdown suppressed activation of MAPK and AKT signaling pathways T24 cells were infected with CCDC34-siRNA lentivirus for 72 hours and subjected to western blot with indicated antibodies. Control, cells infected with NC lentivirus; CCDC34-siRNA, cells infected with CCDC34-siRNA lentivirus. **A.** Effect of CCDC34 knockdown on phosphorylation of P-JNK (JNK/JUN pathway) and P-P38 (P38MAPK pathway). **B.** Effect of CCDC34 knockdown on RAF-MEK-ERK-MAPK pathway. **C.** Effect of CCDC34 knockdown on phosphorylation of AKT. GAPDH served as loading control.

The abnormal expression of the coiled-coil domain containing proteins has a direct link with the phenotype of tumor cell migration, invasion and metastasis, for example CCDC134 is down-regulated in gastric cancer and its silencing promotes the migration and invasion of both the normal gastric epithelial cell line GES-1 and gastric cancer cell line AGS via the MAPK pathway [10]. Amplified proliferation is a characteristic of tumor cells, which is frequently caused by enhanced activity of intracellular signal transduction pathways. In addition, MAPK cascades and AKT pathway are key signaling pathways involved in multiple biologic processes such as cell proliferation, differentiation, death, migration, invasion and inflammation. In order to explore the molecular mechanisms underlying CCDC34 induced cell proliferation and migration, we analyzed the effect of CCDC34 on oncogenic signaling pathways. Western blot results showed that phosphorylation of MEK, ERK1/2, JNK, p38 and AKT were reduced in T24 cells with CCDC34 knockdown; the decreased expression of c-Raf and c-Jun was also observed in the cells (Fig. 6 and Supplemental Fig 2). These results suggested that activation of MAPK and AKT might be responsible for CCDC34 regulation of bladder cancer cell proliferation, apoptosis and migration.

## DISCUSSION

Although there is extensive information from the past about bladder cancer at genetic and molecular level, differing clinical courses and the limited value of established prognostic markers have compelled researchers to look for new molecular parameters in predicting the prognosis and treatment of patients with bladder cancer. Identifying appropriate molecular targets and understanding the molecular basis of these pathways is an important step.

CCDC34 is a protein-coding and disease related gene, while its biological and clinical significances remain largely unknown. During our study, we did a preliminarily functional characterization of CCDC34 expression in bladder carcinomas. Initially, immunohistochemical analysis revealed high expression of CCDC34 in bladder tumors compared with normal urothelium, and Western blot confirmed that CCDC34 was expressed at higher level in human bladder cancer tissues compared with their paraneoplastic bladder tissues. In addition, bladder cancer cell lines 5637, T24 and EJ showed elevated expression of CCDC34, while J82 and BIU-87 did not demonstrate increased CCDC34 expression levels compared with benign cell line SV-HUC-1. The possible reasons for this discrepancy are complicated. This may be due to tumor heterogeneity. In our studies, we chose two different cell lines T24 and 5637 to investigate the biological functions of CCDC34 to rule out the effect of CCDC34 knockdown is just certain cell line specific.

In our study, Lentivirus-mediated CCDC34 knockdown markedly inhibited bladder cancer cell proliferation and migration *in vitro*. The total cell number, cell growth rate and DNA synthesis in CCDC34-siRNA lentivirus infected group were significantly decreased or slowed down compared with the NC lentivirus infected group. Colony formation assay showed CCDC34 knockdown in both T24 and 5637 cells drastically caused a substantial reduction in colony formation numbers. Mouse models bearing T24 cell-derived xenografts were injected with chemically modified CCDC34 siRNA for *in vivo* tumorigenesis assay, and the results showed siCCDC34 treatment retarded tumor growth significantly compared with the siCtrl treatment. Knockdown of CCDC34 also resulted in decreased migration abilities of T24 and 5637 cells. Furthermore, CCDC34 knockdown induced cell cycle arrest at G2/M phase and increased apoptosis. These results demonstrate that CCDC34 plays an important role in bladder cancer cell proliferation and migration, suggesting the oncogenic role of CCDC34 in bladder cancer.

The MAPK pathway comprises several key signaling components and phosphorylation events that play a role in tumor progression and metastasis. There are 5 main subgroups of the MAPKs: ERK (ERK1/ERK2), c-Jun N (JNK/SAPK), p38MAPK (p38α, p38β, p38γ and p38δ), ERK3/ERK4 and ERK5 [17]. ERK1 and ERK2 are key transducers of proliferation, differentiation and survival signals. Activation of the RAS-RAF-MEK-ERK-MAPK pathway has been frequently reported in human cancers [18–19]. Phospho-JNK/SAPK can activate downstream c-Jun, thus regulating cell proliferation and migration. While decreasing p38 activity has much to do with cancer since continuous cell proliferation requires the activity of p38 in most of the cancers studied [4, 20]. Therefore, the phosphorylated expression levels of the MAPK family members (p-JNK, p-p38, p-MEK1/2 and p-ERK1/2) were detected after CCDC34 knockdown and the results showed decreased phosphorylation of these members. c-Jun, as an important signal transduction factor activated by the MAPK family members, was also decreased. Meanwhile, another important signal transduction factor AKT was found to be down-regulated with CCDC34 knockdown. The AKT signaling pathway plays an important regulatory role in many cellular survival pathways, primarily as an inhibitor of apoptosis; besides, it is critical for angiogenesis and tumorigenesis [21]. By activating AKT signaling, the expression of various angiogenic factors [22], angiogenesis [23], and tumor growth [24] is increased. In fact, combined effect of Akt and MARK signal transduction pathways was universal in the action of antitumor agents. So our findings preliminarily declared that CCDC34 silencing may suppress bladder cancer proliferation and migration through inactivation of RAF-ERK1/2-MAPK, p38/MAPK, JNK/MAPK and PI3K/Akt signaling pathways. However, further studies are needed to confirm whether the activation of MAPK or AKT pathways is required for CCDC34-mediated proliferation and migration, e.g. the effect of CCDC34 knockdown plus blocking the MAPK or AKT pathway on bladder cancer cells proliferation, apoptosis and migration; and how CCDC34 regulates the phosphorylation and gene expressions.

Together, our work provided novel information on the function of CCDC34 in bladder cancer cells. However, more mechanisms for CCDC34 function in normal bladder and cancer tissues need further exploration. Elevated CCDC34 was detected in human bladder cancer specimens. But two out of five bladder cancer cell lines did not demonstrate increased CCDC34 expression, and this discrepancy might indicate certain relevance of sensitivity of CCDC34 with individual differences in tumor itself and its malignancy. Next, we would expand the number of our bladder cancer specimens to find whether CCDC34 level is correlated with the tumor grade, pathologic stage and lymph node metastasis to further explore its clinical significance. The detailed mechanisms of CCDC34 in regulating bladder cancer tumorigenesis and progression would also need further studies. Those studies will undoubtedly shed new insights into the use of CCDC34 as a biomarker or even a therapeutic target of bladder cancer.

## MATERIALS AND METHODS

### Tissue samples

Tissue samples were obtained from 42 bladder cancer patients, who underwent radical cystectomy or transurethral resection at Department of Urology, Peking University First Hospital from June 2010 to December 2010. The 42 patients were pathologically confirmed as bladder urothelial carcinoma, including T1 (*n* = 10), T2 (*n* = 11), T3 (*n* = 11), and T4 (*n* = 10). There were 33 men and 9 women, with a mean age of 60 years (range, 22–86). Meanwhile, the normal control bladder epitheliums were obtained from 18 cases among those 42 bladder cancer patients, including T2 (*n* = 6), T3 (*n* = 8) and T4 (*n* = 4), and the normal epitheliums were taken from the area more than 2.0 cm away from the margin of cancer tissue. The histological characteristics of those samples were evaluated by haematoxylin-eosin staining and confirmed by experienced urological pathologists. Fresh samples were fixed in 4% paraformaldehyde for 12–24 h and then paraffin-embedded for immunohistochemistry. A portion of the samples (18 cases) were snap frozen immediately after the resection and stored in liquid nitrogen until use. Informed consent was obtained in all cases and protocols were approved by the Medical Ethics Committee of Peking University First Hospital.

### Cell culture

The normal human urinary tract epithelial cells SV-HUC-1 were obtained from ATCC (Manassas, VA, USA) and cultured in F-12K medium (Hyclone). The bladder cancer cell lines were obtained from the institute of Urology, Peking University; with T24, 5637, BIU-87 and EJ cultured in RPMI-1640 medium (Hyclone), and J82 in high glucose DMEM medium (Hyclone). All media contained 10% fetal bovine serum (GIBCO), penicillin G (100 U/ml), and streptomycin (100 μg/ml) (Sigma-Aldrich). All the cell cultures were maintained as a monolayer culture at 37°C in a humidified atmosphere containing 5% CO_2_.

### Lentivirus vector construction for RNAi

The lentivirus vector system is composed of the vectors pGCSIL-GFP which stably expressed siRNA and a marker (GFP-RFP fusion protein), pHelper1.0 (gag/pol element) and Helper2.0 (VSVG element). The vectors pHelper1.0 and pHelper2.0 contain virus package imperative elements. The most effective double-stranded CCDC34-targeted small RNA interfering sequences, PscSI14255 (5′-TGAAGATGCCCATGATTCA-3′), were synthesized and cloned into the pGCSIL-GFP vector by GeneChem Corporation (Shanghai, China). Psc-NC (5′-TTCTCCGAACGTGTCACGT-3′) was used to generate the negative control lentiviral vectors (NC or control) and showed no homology to any known human genes. Cancer cells were plated in six-well plates (5 × 10^4^ cells/well) until cell fusion reached 60%, and then appropriate volumes of lentivirus were added to the cells according to the MOI value (number of lentiviruses per number of cells) recommended by manufacture.

### Immunohistochemistry

Formalin-fixed, paraffin-embedded tissue sections (5 μm) were deparaffinized in xylene and rehydrated with gradient concentrations of ethanol. Endogenous peroxidase activity was blocked (0.35% H2O2 in PBS buffer), antigens were retrieved by microwaving (350 W), and nonspecific binding was blocked by 1% bovine serum albumin in PBS buffer. Sections were stained with CCDC34 antibody (diluted 1:500; Abcam, ab122396) and visualized with secondary antibody (Envision, DakoCytomation). Slides were then incubated with 3, 3′-diaminobenzidine chromogen (DakoCytomation), counterstained in Meyer's hematoxylin, and mounted with Aquatex (Merck, Darmstadt, Germany).

### Western blot analysis

Whole-cell lysates were prepared with ice-cold radioimmunoprecipitation assay buffer (Sigma-Aldrich), quantified, and loaded onto SDS-PAGE. After electrophoresis, proteins in the gel were transferred to a nitrocellulose membrane and incubated with primary antibodies at 4°C overnight. Western blot was conducted using antibodies specific for CCDC34 (Abcam, ab122396), JUN (Proto-Oncogene C-Jun) (Abcam, ab32137), C-RAF (Cell Signaling Technology, #12552), P-MEK1/2 (Cell Signaling Technology, #9154), ERK1/2(Cell Signaling Technology, #9107), P-ERK1/2(Thr202/Tyr204) (Cell Signaling Technology, #4376), P38(Abcam, ab31828), P-P38 (Cell Signaling Technology, #4631), P-JNK (Thr183/Tyr185) (Cell Signaling Technology, #9251), AKT (Abcam, ab124341), P-AKT(Ser473) (Cell Signaling Technology, #4060) and GAPDH (glyceraldehyde 3-phosphate dehydrogenase) (Santa Cruz Biotechnology, sc-25778), followed with horseradish peroxidase-labeled secondary antibody (Santa Cruz Biotechnology, sc-2004/sc-2005). Signals were detected by chemiluminescence (ECL Western Blotting Detection Reagents, GE Healthcare) and visualized using G:BOX Chemi Gel Documentation System (Syngene, Frederick, MD, USA).

### Cell growth assay

Cell growth was measured via multiparametric high-content screening (HCS). Briefly, three days after T24 cells infected with NC lentivirus or CCDC34-siRNA lentivirus, cells in logarithmic phase were digested, resuspended, counted and inoculated in 96-well plates at 37°C with 5% CO_2_ for 5 days. Plates were processed with the ArrayScan™ HCS software (Cellomics Inc.) for each day's analysis. The system is a computerized, automated fluorescence-imaging microscope that automatically identifies stained cells and reports the intensity and distribution of fluorescence in each individual cell. Images were acquired for each fluorescence channel, using suitable filters and 20 × objective. Images and data were stored in a Microsoft SQL database for easy retrieval.

### BrdU incorporation assay

DNA synthesis in proliferating cells was determined by BrdU incorporation assay, using Brdu kit (Roche, No.11647229001) following manufacturer's instructions.

### Colony formation assay

Cells infected with CCDC34-siRNA lentivirus or NC lentivirus were seeded in 6-well plates at a density of 800 per well and cultured at 37°C for 14 days. Medium was replaced every 2 to 3 days. Cells were washed twice with PBS, fixed with 4% paraformaldehyde, stained with Giemsa for 10 minutes and washed 3 times with double distilled H_2_O. Colonies were photographed and counted under a microscope (Leica DM IL; Leica Microsystems).

### Wound healing assays

The CytoSelect™ 24-well Wound Healing Assay (Cell Biolabs, CBA-120) was used to analyze migration of T24/5637 Control and CCDC34 knockdown cells. The assay was done according to the manufacturer's recommendations using 2.0 × 10^5^ cells per well. Image acquisition of wound fields was done after removal of inserts (0 hours) and wound closure documentation was done after 8 and 24 hours with a phase-contrast microscope (Leica DM IL; Leica Microsystems) equipped with a digital camera (Leica DFC300FX).

### Cell cycle analysis

Cells infected with CCDC34-siRNA lentivirus or NC lentivirus were collected, washed twice with ice-cold phosphatebuffered saline (PBS), and fixed with 70% ice-cold ethanol. After fixation overnight and subsequent rehydration in PBS for 30 min at 4°C, the samples were stained for 30 min in dark with 50 g/ml propidium iodide (Sigma-Aldrich, P4170) containing 125 U/ml protease-free RNase, and then analyzed using a flow cytometer (FACSCalibur, Becton Dickinson). Cell cycle analysis was carried out using ModFit 2.0 software (Becton Dickinson).

### Cell apoptosis analysis

Cell apoptosis was assayed by staining with Annexin V-APC (ebioscience, 88–8007) following manufacturer's instructions and detected by a flow cytometer (FACSCalibur, Becton Dickinson).

### Animal experiments

Four-week-old male/female BALB/c nude mice were injected subcutaneously in the right flank with 3 × 10^6^ T24 cells. After 7 days, the mice were randomly selected for treatment with CCDC34 siRNA (6) or as controls (6). The chemically modified, cholesterol-conjugated CCDC34 siRNA and corresponding negative control (siCtrl) for *in vivo* RNA delivery were from Ribobio Co. (Guangzhou, China) [25–26]. 10 nmol RNA in 50 μl saline buffers was locally injected into the tumor mass twice a week for 4 weeks. Tumor size was measured 2 times each week and calculated using the formula: (length × width^2^)/2. All animal procedures were conducted in accordance with the policies and regulations of Peking University Institutional Animal Care and Use Committee (Beijing, China).

### Statistical analysis

Student's *t*-test and Kruskal-Wallis test were used for raw data analysis. Statistical analysis was performed using the SPSS16.0 software package. All values in the text and figures are expressed as the mean ± SD of these observations. Statistical significance was considered at *P*-value < 0.05.

## SUPPLEMENTARY FIGURES


