# Supplementary material for: CCDC34 is up-regulated in bladder cancer and regulates bladder cancer cell proliferation, apoptosis and migration
Source: Oncotarget. 2015 Jul 16;6(28):25856–67. doi: 10.18632/oncotarget.4624 (PMC4694871; doi:10.18632/oncotarget.4624)
Supplement: Supplementary file 1 [file oncotarget-06-25856-s001.pdf]

## SUPPLEMENTARY FIGURES

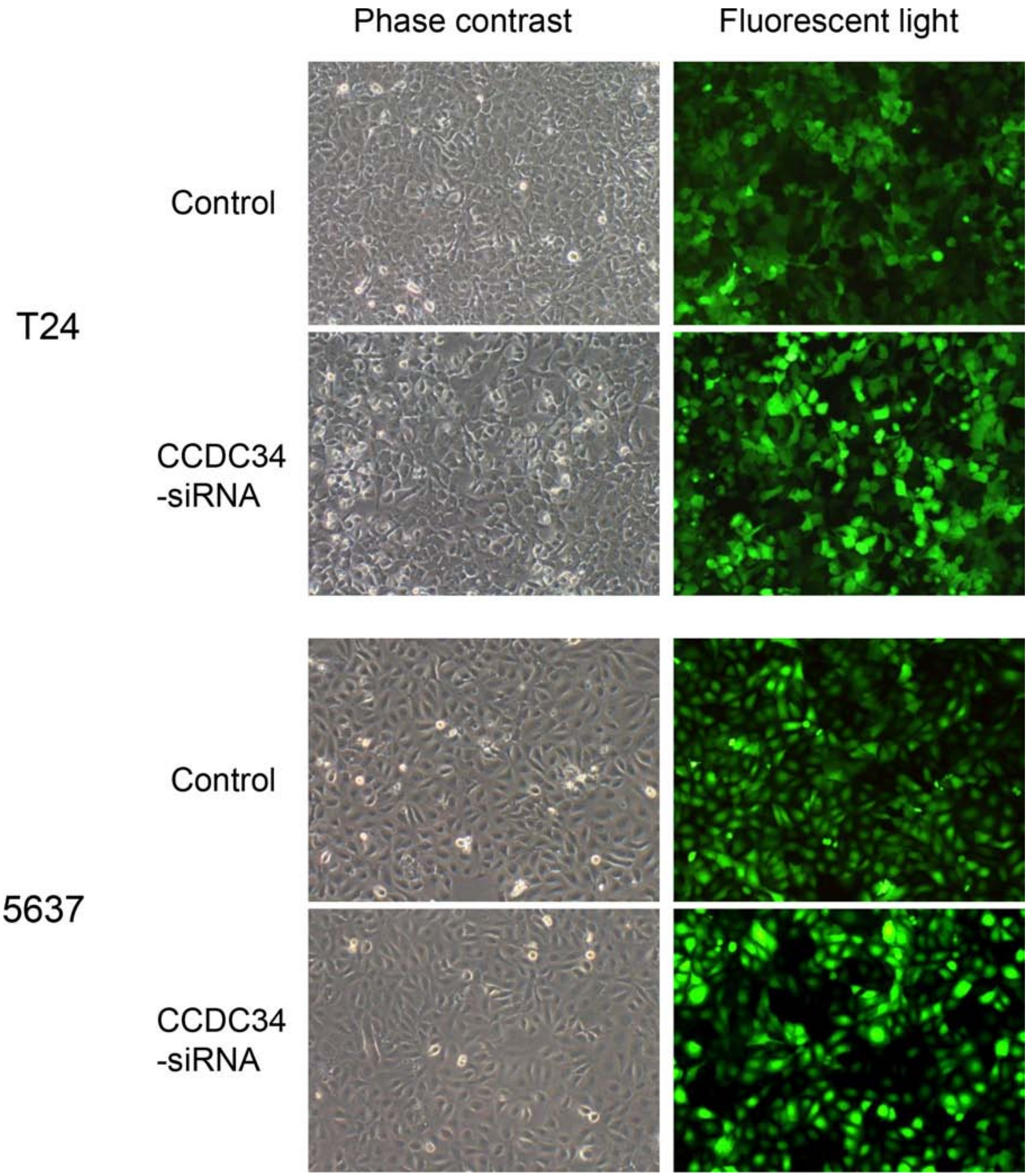


**Supplementary Figure S1: The efficiency of lentivirus infection.** Bladder cancer cell lines T24 and 5637 were infected with CCDC34-siRNA or NC lentivirus, and examined by fluorescent microscopy and light microscopy at the 3rd day after infection. Representative images of the cultures are shown. More than 85% of the cells expressed GFP (magnified 200X)

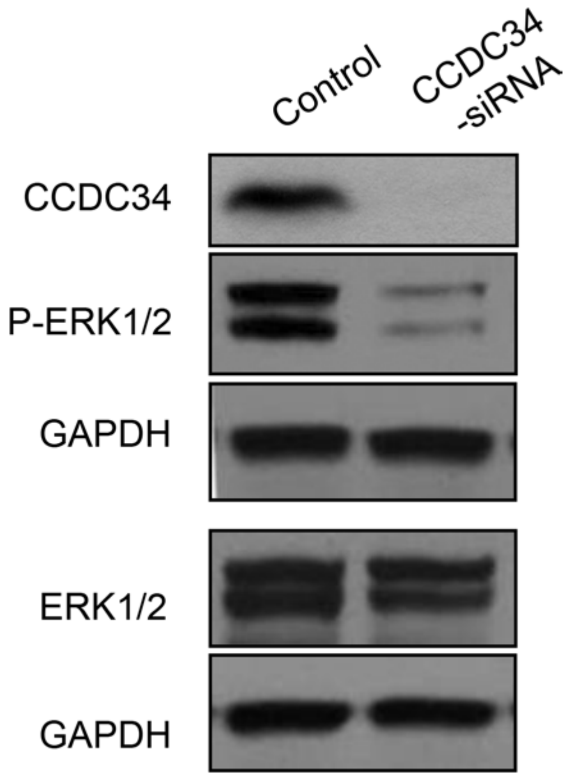


**Supplementary Figure S2: Effect of CCDC34 knockdown on ERK1/2 and P-ERK1/2.** T24 cells were infected with CCDC34-siRNA lentivirus for 48 hours and subjected to western blot with indicated antibodies.
